# Tumor microenvironment delineates differential responders to trastuzumab emtansine in HER2-positive metastatic breast cancer patients previously treated with pyrotinib: an exploratory biomarker analysis of a phase II study (NJMU-BC02)

**DOI:** 10.1038/s41392-025-02409-2

**Published:** 2025-09-29

**Authors:** Hong Pan, Ji Wang, Yue Sun, Fanfan Li, Chang Sun, Mingduo Liu, Hong Xu, Jing Tao, Xinrui Mao, Cong Wang, Shui Wang, Wei Li, Qiang Ding, Wenbin Zhou

**Affiliations:** 1 Department of Breast Surgery, Department of General Surgery, The First Affiliated Hospital with Nanjing Medical University, Nanjing, China; 2 Jiangsu Key Lab of Cancer Biomarkers, Prevention and Treatment, Jiangsu Collaborative Innovation Center for Cancer Personalized Medicine, School of Public Health, Nanjing Medical University, Nanjing, China; 3 Department of Oncology, The First Affiliated Hospital with Nanjing Medical University, Nanjing, China; 4 Department of Oncology, The Second Affiliated Hospital of Anhui Medical University, Hefei, China; 5 Department of Medical Oncology, The First Affiliated Hospital of Soochow University, Suzhou, China; 6 Department of General Surgery, The Fourth Affiliated Hospital of Nanjing Medical University, Nanjing, China; 7 Department of Pathology, The First Affiliated Hospital with Nanjing Medical University, Nanjing, China

**Keywords:** Breast cancer, Cancer microenvironment, Predictive markers, Tumour heterogeneity, Tumour immunology

## Abstract

Trastuzumab emtansine (T-DM1) has been approved for the treatment of HER2-positive breast cancer. However, the efficacy of T-DM1 for patients after failure of pyrotinib and/or trastuzumab plus pertuzumab has not been clear. Additionally, no biomarker has been reported to predict the effect of T-DM1. In this multicenter phase II trial (NCT06125834), 36 participants with HER2-positive metastatic breast cancer were enrolled to receive T-DM1 therapy on a 21-day cycle until progression or unacceptable toxicity. The primary endpoint was the objective response rate (ORR). The secondary endpoints included the disease control rate (DCR), clinical benefit rate (CBR), progression-free survival (PFS), and toxicity. The primary endpoint was an ORR of 47.2% (17/36, 95% CI 30.4–64.5). The treatment exhibited a manageable toxicity profile. The DCR was 66.7% (24/36, 95% CI 49.0–81.4), and the CBR was 50.0% (18/36, 95% CI 32.9–67.1). The median PFS was 6.6 (95% CI 5.2-NA) months. Single-cell RNA sequencing revealed that the low cell cycle activity of cancer cells, activated macrophages and CD8+ T cells was associated with the good efficacy of T-DM1, which was validated in a neoadjuvant cohort. This study suggests that T-DM1 is effective with a measurable safety profile in patients with metastatic HER2-positive breast cancer after failure of pyrotinib and/or trastuzumab plus pertuzumab. Our preliminary findings suggest potential biomarkers that may help predict T-DM1 efficacy, generating hypotheses for novel therapeutic targets that may address T-DM1 resistance.

## Introduction

Human epidermal growth factor receptor 2 (HER2)-positive breast cancer accounts for ~15–20% of all breast cancer cases and is characterized by high malignancy and poor prognosis until the introduction of HER2-targeted therapies.^1^ Anti-HER2 therapy significantly improves the survival of these patients.^2,3^ With the continuous progress of medical research, anti-HER2 therapeutic drugs, including monoclonal antibodies, tyrosine kinase inhibitors (TKIs), and antibody-conjugated drugs (ADCs), continue to emerge. For patients with HER2-positive metastatic breast cancer, the use of dual anti-HER2 components directed at HER2 with complementary mechanisms of action can provide a more comprehensive blockade of HER2 signaling than the use of either agent alone.^4^ In the first-line setting, the combination of two anti-HER2 monoclonal antibodies (trastuzumab and pertuzumab) with docetaxel has been accepted as the standard of care for metastatic HER2-positive breast cancer as a result of the CLEOPATRA study.^5,6^ Pyrotinib is a small-molecule, irreversible, pan-HER receptor TKI that targets EGFR, HER2, and HER4.^7^ Pyrotinib plus capecitabine has been approved for clinical use as a second-line treatment for HER2-positive metastatic breast cancer in China.^8^ Additionally, the PHILA study^4^ has revealed that pyrotinib, trastuzumab and docetaxel significantly improve progression-free survival (PFS) compared with placebo, trastuzumab and docetaxel in patients with untreated HER2-positive metastatic breast cancer, although grade ≥3 diarrhea occurred in 46% of patients. The combination of pyrotinib with trastuzumab and docetaxel has been granted approval as a first-line treatment for HER2-positive advanced breast cancer in China.

In the second-line setting, trastuzumab emtansine (T-DM1) after trastuzumab-based therapy has been the preferred regimen recommended by international treatment guidelines.^9,10^ The DESTINY-Breast03 study^11–13^ has revealed that trastuzumab deruxtecan (T-DXd) has superior efficacy over T-DM1, and T-DXd has been the first choice for second-line treatment internationally. T-DXd has not yet entered medical insurance in China and is expensive. T-DM1 is still widely accepted as a second-line treatment for advanced HER2-positive breast cancer in clinical practice. Even after the failure of widely used pyrotinib and/or trastuzumab plus pertuzumab, the efficacy of T-DM1 is still not very clear,^14^ although T-DXd may be the best choice.

Drug resistance limits the use of T-DM1 in the treatment of breast cancer. The mechanisms of ADC resistance can be summarized into four categories: antibody-mediated resistance, impaired drug trafficking, disrupted lysosomal function, and payload-related resistance.^15^ Previous studies have shown that the mRNA expression of HER2, HER3, or *PIK3CA* is not associated with the effect of T-DM1.^16,17^ To the best of our knowledge, no single accurate molecular marker has been able to fully predict the efficacy of T-DM1.

In current clinical practice, there is an urgent need to clarify the efficacy and safety of T-DM1 in patients resistant to pyrotinib and trastuzumab plus pertuzumab and to explore molecular markers that can predict efficacy to guide clinical practice to maximize the efficacy of T-DM1 and the quality of life of patients. The NJMU-BC02 study (NCT06125834), a phase II study, assessed the efficacy and safety of T-DM1 in patients with HER2-positive metastatic breast cancer previously treated with pyrotinib and/or trastuzumab plus pertuzumab. Single-cell RNA sequencing was used to identify markers to predict the effect of T-DM1.

## Results

### Study population

**Fig. 1:**
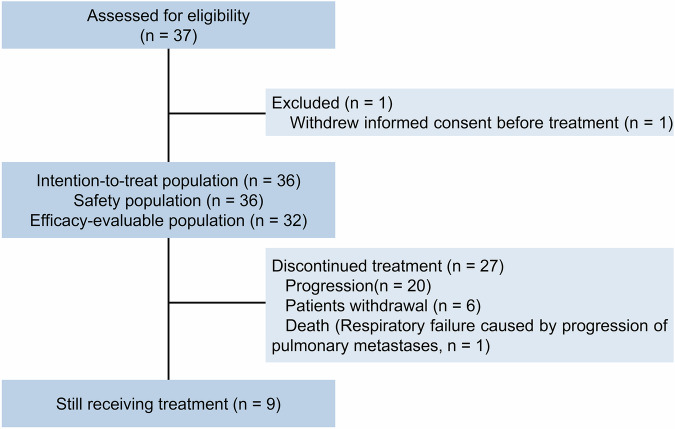
Flow chart of the NJMU-BC02 study

From October 2023 through March 2025, a total of 36 participants were enrolled at 4 academic hospitals in China (Fig. 1). Overall, the responses of 32 (88.9%) participants were evaluable. As of the cutoff date of March 1, 2025, the median follow-up time was 12.8 (range, 0.7–15.9) months. At the time of analysis, one (2.8%) of the 36 participants had died due to respiratory failure caused by the progression of pulmonary metastases, and nine (25.0%) were still receiving treatment (Fig. 1). Among all the enrolled participants, 12 (33.3%) participants received one line, and 20 (55.6%) received at least two lines of treatment in the advanced setting before enrollment (Table 1). Thirty-four (94.4%) patients received trastuzumab, 25 (69.4%) patients received pertuzumab, and 23 (63.9%) patients received pyrotinib prior to enrollment. Additionally, 16 (44.4%) participants previously received both pyrotinib and trastuzumab plus pertuzumab (Table 1).

**Table 1:** Clinicopathological features of the enrolled participants

	T-DM1 (*n* = 36)
Age, median, (range), years	57.5 (23~88)
Female, *n* (%)	36 (100)
HER2 status (IHC), *n* (%)	
3+	27 (75.0)
2+ (FISH amplified)	9 (25.0)
ECOG PS, *n* (%)	
0	24 (66.7)
1	12 (33.3)
Hormone receptor, *n* (%)	
Positive	16 (44.4)
Negative	19 (52.8)
NE	1 (2.8)
Prior treatment for mBC, *n* (%)	
No	4 (11.1)
Yes	32 (88.9)
Metastatic sites, *n* (%)	
Visceral	30 (83.3)
Nonvisceral	5 (13.9)
NE	1 (2.8)
Prior lines of therapy in the metastatic setting, *n* (%)	
0	4 (11.1)
1	12 (33.3)
2	17 (47.2)
≥ 3	3 (8.4)
Disease-free interval of trastuzumab treatment, *n* (%)	
< 1 year	12 (33.3)
≥ 1 year	24 (66.7)
Prior cancer therapy, *n* (%)	
Trastuzumab	34 (94.4)
Pertuzumab	25 (69.4)
TKI	23 (63.9)
ADC	1 (2.8)

*T-DM1* trastuzumab emtansine, *HER2* human epidermal growth factor receptor 2, *IHC* immunohistochemistry, *FISH* fluorescence in situ hybridization, *ECOG* *PS* Eastern Cooperative Oncology Group performance status, *NE* not evaluable, *mBC* metastatic breast cancer, *TKI* tyrosine kinase inhibitors, *ADC* antibody-conjugated drugs

### Efficacy

**Fig. 2:**
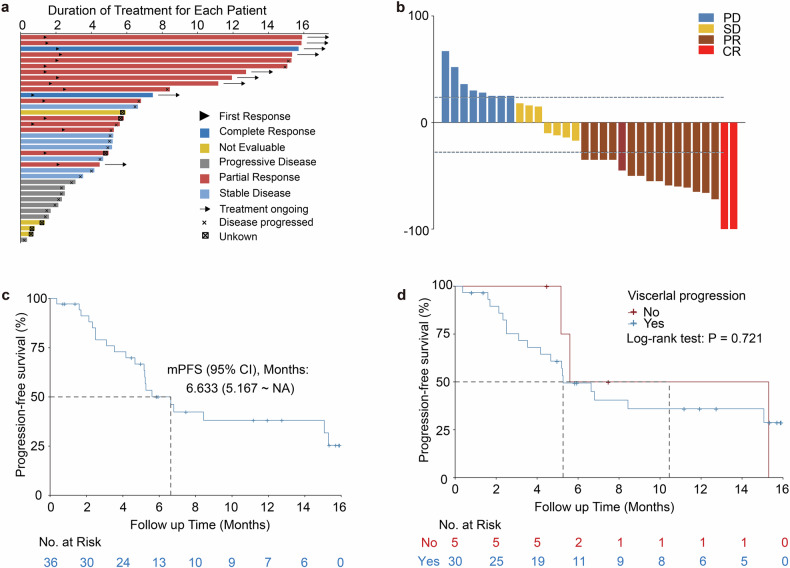
Efficacy of trastuzumab emtansine in the enrolled participants. **a**, **b** The primary endpoint of the NJMU-BC02 study according to RECIST 1.1. **c** PFS of all enrolled participants. **d** PFS of enrolled participants with or without visceral progression. Significance was determined by the log-rank test (**d**)

The efficacy of T-DM1 was analyzed in all participants. The primary endpoint of this trial was met (Fig. 2a, b and Table 2). The objective response rate (ORR) was 47.2% (17/36, 95% CI 30.4–64.5). Two (5.6%) and 15 (41.7%) participants had a best response of complete response (CR) or partial response (PR), respectively. Additionally, 7 (19.4%) participants had stable disease (SD), and 8 (22.2%) had progressive disease (PD). The disease control rate (DCR) was 66.7% (24/36, 95% CI 49.0–81.4), and the clinical benefit rate (CBR) was 50.0% (18/36, 95% CI 32.9–67.1) (Fig. 2a, b and Table 2).

**Table 2:** Efficacy of trastuzumab emtansine in the enrolled participants

	Intention-to-treat population (*n* = 36)	Efficacy-evaluable population (*n* = 32)
Best overall response		
CR, *n* (%)	2 (5.6)	2 (6.3)
PR, *n* (%)	15 (41.7)	15 (46.9)
SD, *n* (%)	7 (19.4)	7 (21.9)
PD, *n* (%)	8 (22.2)	8 (25.0)
NE, *n* (%)	4 (11.1)	NA
ORR, *n* (%)	17 (47.2)	17 (53.1)
DCR, *n* (%)	24 (66.7)	24 (75.0)
CBR, *n* (%)	18 (50.0)	18 (56.3)

*CR* complete response, *PR* partial response, *SD* stable disease, *PD* progressive disease, *NE* not evaluable, *NA* not applicable, *ORR* objective response rate, *DCR* disease control rate, *CBR* clinical benefit rate

After a median follow-up of 12.8 (range, 0.7–15.9) months, the median PFS was 6.6 (95% CI 5.2-NA) months (Fig. 2c), and the median overall survival (OS) was not reached. There was no significant difference in PFS between patients with and without visceral progression (log-rank test, *P* = 0.721) (Fig. 2d). Participants with different lines of prior therapies in the advanced setting (≤1 line vs >1 line) had similar ORRs, DCRs, CBRs and median PFS rates (Supplementary Table 1). Two CR cases (one with supraclavicular lymph node metastases and another with liver metastases) were found among participants with ≤1 line of prior therapy in the advanced setting (Supplementary Table 1). Therefore, T-DM1 demonstrated favorable efficacy in HER2-positive metastatic breast cancer patients, meeting the study’s predefined endpoints.

### Safety

We evaluated the safety profile of T-DM1 in all participants. Twenty-five of the 36 participants experienced treatment-related adverse events of any grade (Supplementary Table 2). The most common treatment-related adverse events were thrombocytopenia (30.6%), diarrhea (22.2%), nausea (19.4%), neutropenia (16.7%), and vomiting (11.1%). Grade 3/4 treatment-related adverse events, including thrombocytopenia (11.1%), diarrhea (2.8%), anemia (2.8%), increased alanine aminotransferase (2.8%), and increased aspartate aminotransferase (2.8%), occurred in 5 participants. Treatment interruption was observed in two patients because of thrombocytopenia. No treatment-related deaths occurred. Therefore, T-DM1 demonstrated a manageable toxicity in HER2-positive metastatic breast cancer patients.

### Single-cell landscape of tumor samples

**Fig. 3:**
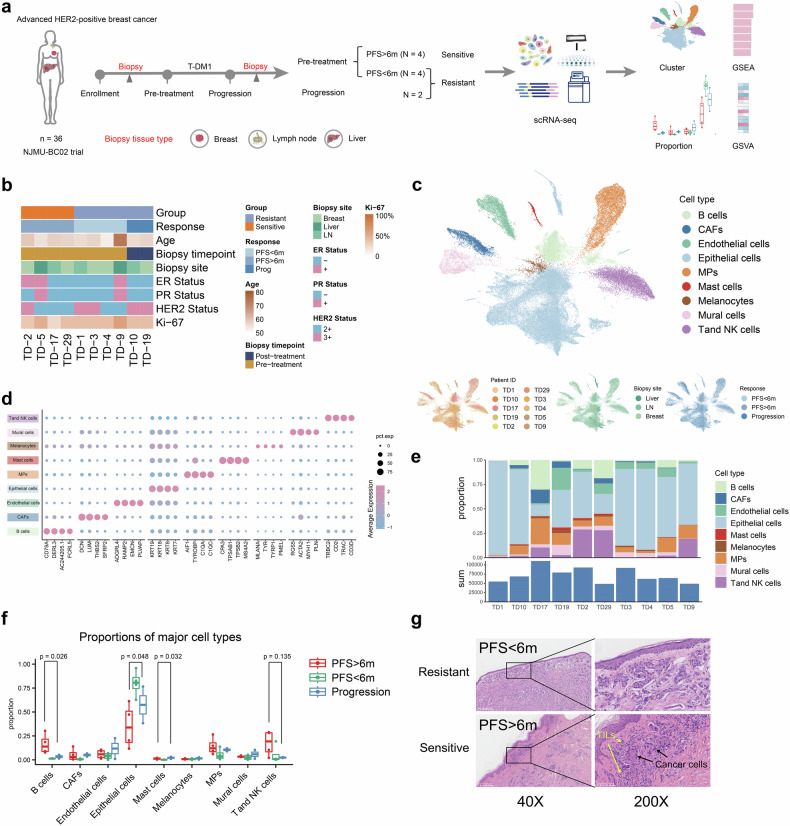
Landscape of tumor samples from 10 participants via single-cell RNA sequencing. **a** Design of the NJMU-BC02 trial in the treatment of advanced HER2-positive breast cancer patients. A total of 8 pretreatment biopsy samples and 2 samples collected at the time of disease progression were assembled for single-cell RNA sequencing analysis. **b** Summary of the clinical characteristics of patients subjected to biopsy. **c** Uniform manifold approximation and projection (UMAP) plot of 80,239 cells colored by cell type, patient ID, biopsy site, and response. **d** Dot plot of the expression of genes defining major cell types. **e** Bar plots of proportions of cell types by patient, with a display of the total cell count for each patient. **f** Box plots showing the differences in the proportions of major cell types. **g** Representative H&E-stained sections of the tumors in (**a**). Data are represented as the median and interquartile range (**f**). Significance was determined by the Kruskal‒Wallis test (**f**). Significance was determined as *P* < 0.05. Parts of (**a**) were drawn from pictures from Servier Medical Art. Servier Medical Art by Servier is licensed under a Creative Commons Attribution 3.0 Unported License (https://creativecommons.org/licenses/by/3.0/)

Exploratory analyses were performed to investigate biomarkers associated with T-DM1 efficacy. Tumor samples from 8 participants before treatment and 2 participants with progressive disease after T-DM1 were available for single-cell RNA sequencing to identify potential biomarkers (Fig. 3a, b). Among these 8 participants, 4 had a PFS > 6 months, recognized as sensitive cases, and another 4 had rapid progressive disease within 6 months.

After standard data processing and quality control procedures, 80,239 scRNA-seq profiles were classified (Fig. 3c, d), including 3191 fibroblasts, 4984 endothelial cells, 43,995 epithelial cells, 831 mast cells, 857 melanocytes, 3092 mural cells, 8648 mononuclear phagocytes, 7141 B cells, and 7500T and NK cells. The cell types among the different biopsy sites, participants, and different treatment responses were similar (Fig. 3c, e). Among the three groups, patients with a PFS > 6 months had the lowest proportion of epithelial cells (*P* = 0.048), the highest proportion of B cells (*P* = 0.026), and the lowest proportion of T and NK cells (*P* = 0.135) (Fig. 3f). Patients with a PFS < 6 months presented similar cell distributions to those with progressive disease (Fig. 3f). Accordingly, four patients with a PFS < 6 months and two patients with progressive disease were recognized as resistant patients. Sensitive cases presented more tumor-infiltrating lymphocytes than did resistant cases, as confirmed by hematoxylin‒eosin (H&E) staining (Fig. 3g).

### High cell cycle activity in cancer cells is indicative of T-DM1 resistance

**Fig. 4:**
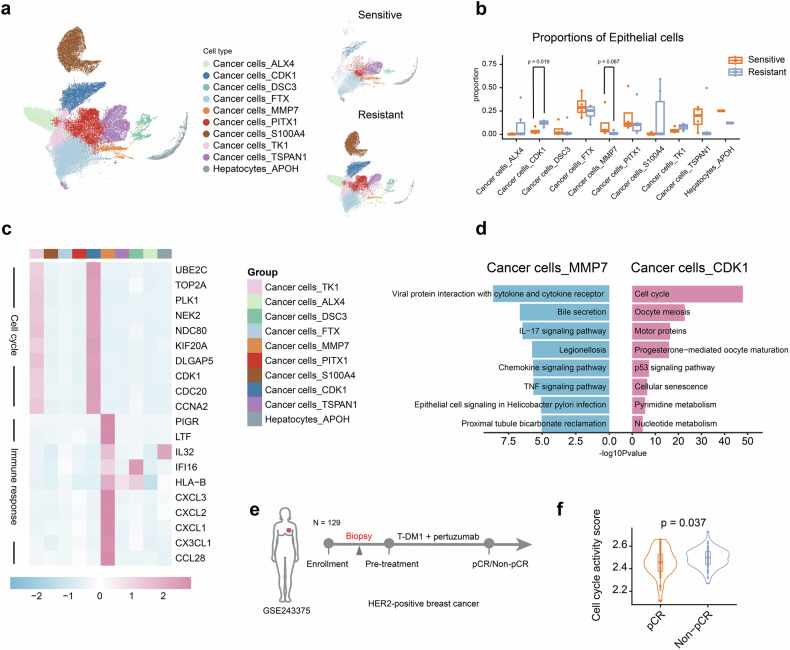
Cell cycle activity of cancer cells can predict the efficacy of trastuzumab emtansine treatment in HER2-positive metastatic breast cancer. **a** UMAP plot of epithelial cells colored by cell cluster; UMAP also shows the distinction between the sensitive and resistant groups. **b** Box plots showing the differences in the proportions of epithelial cells. **c** Heatmap showing the expression of genes associated with the cell cycle and immune response in epithelial cells. **d** Bar plots showing the mean scores of KEGG pathways related to cancer cell_CDK1 and cancer cell_MMP7. **e** Design of the clinical trial related to GSE243375 in the GEO database. A total of 129 pretreatment biopsy samples from patients with HER2-positive breast cancer were assembled for RNA sequencing analysis. **f** Violin plots showing the cell cycle activity scores of the tumor samples in (**e**), which were calculated via ssGSEA. Data are represented as the median and interquartile range (**b**, **f**). Significance was determined by the Wilcoxon rank-sum test (**b**) or two-tailed unpaired *t* test (**f**). Significance was determined as *P* < 0.05

To determine the underlying mechanisms of T-DM1 resistance, 10 subgroups of epithelial cells were identified (Fig. 4a and Supplementary Fig. 1a). Compared with the resistant cases (Fig. 4b), the sensitive cases presented a significantly lower proportion of cancer cells expressing CDK1 (*P* = 0.019) and a trend toward a greater proportion of cancer cells expressing MMP7 (*P* = 0.067). The proportions of cancer cells_CDK1 and cancer cells_MMP7 were similar between the “PFS < 6 m” and “progression” groups (Supplementary Fig. 1b). Among these 10 subgroups of epithelial cells, cancer cells_CDK1 were characterized by high expression of UBE2C, CDK1, TOP2A, PLK1, NEK2, NDC80, KIF20A, DLGAP5, CDC20, and CCNA2 (Fig. 4c), indicating high cell cycle activity. Additionally, gene ontology (GO) enrichment analysis and Kyoto Encyclopedia of Genes and Genomes (KEGG) analysis revealed that genes associated with the cell cycle, chromosome segregation, nuclear division, mitotic nuclear division, and microtubule cytoskeleton organization involved in mitosis were enriched in cancer cells_CDK1 (Fig. 4d and Supplementary Fig. 1c). Similarly, cancer cells_TK1 also exhibited cell cycle activity (Fig. 4c), with a trend toward a greater proportion of resistant cases. According to previous preclinical studies^18,19^ and our data, cancer cells_CDK1 may be resistant to T-DM1. Cancer cell_MMP7 was characterized by high expression of MMP7, PIGR, LTF, HLA-B, CXCL3, CXCL2, CXCL1, CX3CL1, and CCL28 (Fig. 4c). GO enrichment analysis and KEGG analysis indicated that cancer cells_MMP7 were enriched in genes associated with humoral immune response, IL-17 signaling pathway, chemokine signaling pathway, and TNF signaling pathway (Fig. 4d and Supplementary Fig. 1c), suggesting that cancer cells_MMP7 may be associated with the immune response.

The mRNA levels of HER2 and HER3 may be involved in resistance to HER2-directed therapies.^17^ To investigate other potential mechanisms, HER2 and HER3 mRNA levels were compared between the two groups. Both HER2 and HER3 mRNA levels were significantly higher in resistant cancer cells than in sensitive cells (Supplementary Fig. 1d). In addition, HER2 and HER3 mRNA levels in tumor cells exhibited similar expression patterns between the “PFS < 6 m” and “progression” groups (Supplementary Fig. 1e). These results suggest that HER2 and HER3 mRNA levels are not involved in resistance to T-DM1.

To further validate the role of cell cycle activity in T-DM1 resistance, a neoadjuvant cohort^20^ with 129 patients receiving T-DM1 plus pertuzumab was used (Fig. 4e). The cell cycle activity score was calculated by using the mRNA levels of cell cycle-associated genes (Supplementary Table 3) in tumor tissues before neoadjuvant therapy. The cell cycle activity score in the pCR group was significantly lower than that in the non-pCR group (Fig. 4f). The gene set enrichment score related to cancer cells_MMP7 was calculated, and there was no significant difference in the score between the pCR and non-pCR groups (Supplementary Fig. 1f). The above results suggest that cell cycle activity can predict the effect of T-DM1 in the treatment of HER2-positive breast cancer.

### Activated CD8+ T cells are associated with increased T-DM1 sensitivity

**Fig. 5:**
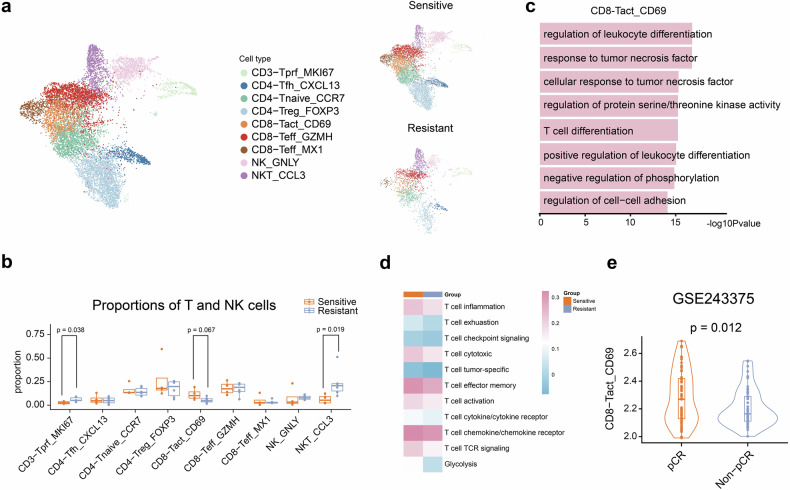
A signature of early activated CD8-Tact-CD69 cells can predict the efficacy of trastuzumab emtansine treatment in HER2-positive metastatic breast cancer. **a** UMAP plot of T and NK cells colored by cell cluster; UMAP also shows the distinction between the sensitive and resistant groups. **b** Box plots showing the differences in the proportions of T and NK cells. **c** Bar plots showing the mean scores of GO pathways related to CD8-Tact-CD69. **d** Heatmap of gene set enrichment scores for CD8+ T cells calculated via ssGSEA. **e** Violin plots showing the gene set enrichment score related to CD8-Tact-CD69, which is based on RNA-seq data from GSE243375 and was calculated via ssGSEA. The data are presented as medians and interquartile ranges (**b**, **e**). Significance was determined by the Wilcoxon rank-sum test (**b**, **d**) or two-tailed unpaired *t* test (**e**). Significance was determined as *P* < 0.05

CD8+ T cells play crucial roles in the antitumor immune response. For T and NK cells, 9 clusters were identified (Fig. 5a and Supplementary Fig. 2a), including CD3-Tprf-MKI67, CD4-Tfh-CXCL13, CD4-Tnaive-CCR7, CD4-Treg-FOXP3, CD8-Tact-CD69, CD8-Teff-GZMH, CD8-Teff-MX1, NK-GNLY, and NKT-CCL3. Compared with that in resistant patients (Fig. 5b), the proportion of CD8-Tact-CD69+ cells was marginally greater in sensitive patients (*P* = 0.067). The proportions of CD8-Tact-CD69+ cells were similar between the “PFS < 6 m” and “progression” groups (Supplementary Fig. 2b). Similar proportions of other subtypes of T and NK cells were observed between the two groups (Fig. 5b). The early activated CD8-Tact-CD69 cells were enriched in genes associated with mononuclear cell differentiation, T-cell differentiation, regulation of leukocyte differentiation, response to tumor necrosis factor, the IL-17 signaling pathway, and lymphocyte differentiation (Fig. 5c and Supplementary Fig. 2c).

The biological functions of the CD8+ T cells were calculated (Fig. 5d and Supplementary Fig. 2d). The biological functions of sensitive cases, including T-cell activation, cytotoxic function, T-cell effector memory, and cytokine function, were significantly greater than those of resistant cases. The functional assessment of CD8+ T cells revealed comparable profiles between the “PFS < 6 m” and “progression” groups (Supplementary Fig. 2e). These results suggest a better antitumor CD8+ T-cell immune response in sensitive patients. Additionally, CD4+ T cells in sensitive patients presented greater immune activity than did those in resistant patients (Supplementary Fig. 2f). The functional assessment of CD4+ T cells revealed comparable profiles between the “PFS < 6 m” and “progression” groups (Supplementary Fig. 2g). The above findings suggest that sensitive patients had higher T-cell immune activity than resistant patients do.

To validate the above results, the signature of CD8-Tact-CD69 was calculated in the neoadjuvant cohort. The signature of CD8-Tact-CD69 in the pCR group was significantly greater than that in the non-pCR group (*P* = 0.012, Fig. 5e). The above results suggest that the CD8-Tact-CD69 signature can predict the effect of T-DM1 in the treatment of HER2-positive breast cancer.

### The FOLR2- and IL-1B-related macrophage gene signatures are predictive of favorable responses to T-DM1

**Fig. 6:**
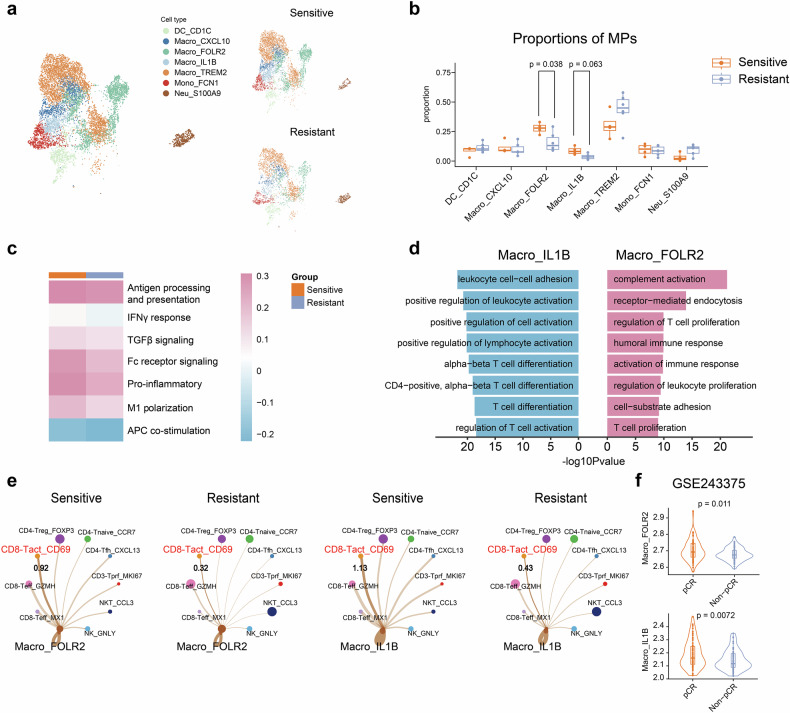
Signatures of Macro-FOLR2 and Macro-IL-1B cells can predict the efficacy of trastuzumab emtansine treatment in HER2-positive metastatic breast cancer. **a** UMAP plot of MPs colored by cell cluster; UMAP also shows the distinction between the sensitive and resistant groups. **b** Box plots showing the differences in the proportions of MPs. **c** Heatmap of gene set enrichment scores for macrophages calculated via ssGSEA. **d** Bar plots showing the mean scores of GO pathways related to macro-FOLR2 and macro-IL-1B. **e** Circle plots showing differential interaction strengths between macro-FOLR2 or macro-IL-1B and T and NK cells. **f** Violin plots showing the gene set enrichment scores related to macro-FOLR2 and macro-IL-1B, which were calculated via ssGSEA on the basis of RNA-seq data from GSE243375. The data are presented as the median and interquartile range (**b**, **f**). Significance was determined by the Wilcoxon rank-sum test (**b**) or two-tailed unpaired *t* test (**f**). Significance was determined as *P* < 0.05

Mononuclear phagocytes play key roles in driving the regulation of T cells, and 7 clusters were identified (Fig. 6a and Supplementary Fig. 3a), including DC-CD1c, Macro-CXCL10, Macro-FOLR2, Macro-IL1B, Macro-TREM2, Mono-FCN1, and Neu-S100A9. Compared with resistant cases (Fig. 6b), a significantly greater proportion of sensitive cases were Macro-FLOR2 (*P* = 0.038), and a marginally greater proportion of sensitive cases were Macro-IL1B (*P* = 0.063). The proportions of macro-FLOR2 and macro-IL-1B were similar between the “PFS < 6 m” and “progression” groups (Supplementary Fig. 3b). Therefore, macrophages may be important in the regulation of the antitumor immune response in this study, and the biological functions of macrophages were compared between the two groups. Compared with resistant cases, sensitive cases presented greater biological activity (Fig. 6c), including antigen processing and presentation signatures, IFN-γ responses, TGF-β signaling, Fc receptor signaling, proinflammatory, M1 polarization, and APC costimulation. The functional assessments of macrophages revealed comparable profiles between the “PFS < 6 m” and “progression” groups (Supplementary Fig. 3c). GO analysis indicated that Macro-FOLR2 and Macro-IL1B were enriched in genes associated with leukocyte cell–cell adhesion, positive regulation of lymphocyte activation, T-cell proliferation, T-cell differentiation, and other processes (Fig. 6d). Additionally, enhanced adhesion interactions between macro-FOLR2 or macro-IL-1B and activated and effective CD8+ T cells were observed in sensitive patients compared with resistant patients (Fig. 6e and Supplementary Fig. 3d, e). The adhesion interactions between macro-FOLR2 or macro-IL-1B and activated and effective CD8+ T cells were comparable between the “PFS < 6 m” and “progression” groups (Supplementary Fig. 3f). The above results suggested that macro-FOLR2 and macro-IL-1B might contribute to the enhanced activated CD8+ T-cell response.

To validate the above results, the signatures of Macro-FOLR2 and Macro-IL-1B were calculated in the neoadjuvant cohort. The signatures of Macro-FOLR2 and Macro-IL1B in the pCR group were significantly greater than those in non-pCR group (both *P* < 0.05, Fig. 6f). The above results suggested that Macro-FOLR2 and Macro-IL1B signatures could predict the effect of T-DM1 in the treatment of HER2-positive breast cancer.

## Discussion

T-DM1 is widely accepted for the treatment of metastatic HER2-positive breast cancer in clinical practice. However, the efficacy of T-DM1 for patients after failure of pyrotinib or other licensed TKIs and/or trastuzumab combined with pertuzumab is not very clear. In the TH3RESA study,^21^ patients with HER2-positive metastatic breast cancer previously treated with trastuzumab and lapatinib (advanced setting) and a taxane (any setting) demonstrated significantly prolonged overall survival with T-DM1 compared with the physician’s choice of treatment (22.7 months vs 15.8 months). The KAMILLA study^22^ enrolled 126 HER2-positive breast cancer patients with measurable brain metastases who had previously received anti-HER2 therapy and/or chemotherapy. Treatment with T-DM1 demonstrated an ORR of 21.4% and a CBR of 42.9%. Additionally, no special biomarker has been reported to predict the effect of T-DM1. In our trial, T-DM1 was effective for treating HER2-positive breast cancer after the failure of pyrotinib and/or trastuzumab plus pertuzumab, with an ORR of 47.2% and a manageable toxicity profile. To the best of our knowledge, biomarkers to predict the efficacy of T-DM1 have been reported for the first time. The low cell cycle activity of cancer cells and activated signatures of macrophages and CD8+ T cells are related to the promising effect of T-DM1 in the treatment of HER2-positive breast cancer, providing a treatment target to reverse resistance to T-DM1.

Dual anti-HER2 therapy has been widely used in adjuvant and advanced settings. For first-line treatment of HER2-positive metastatic breast cancer, trastuzumab combined with pertuzumab or pyrotinib has been approved in China. Additionally, pyrotinib plus capecitabine has also been approved for second-line therapy after failure of trastuzumab. In the HER2CLIMB study,^23^ the addition of tucatinib to trastuzumab and capecitabine significantly improved PFS from 5.6 months to 7.8 months in patients with HER2-positive metastatic breast cancer previously treated with trastuzumab, pertuzumab and T-DM1. In the NALA study,^24^ neratinib combined with capecitabine demonstrated a greater PFS benefit than lapatinib plus capecitabine in patients with metastatic HER2-positive breast cancer previously treated with ≥2 HER2-targeted therapies. T-DXd has been accepted as standard second-line therapy for HER2-positive metastatic breast cancer. T-DM1 is still used in clinical practice, although it is most commonly used after T-DXd where available. However, the efficacy of pyrotinib in T-DM1 patients after failure has not been reported. In the EMILIA study,^9^ T-DM1 demonstrated superior efficacy overlapatinib plus capecitabine in patients with HER2-positive advanced breast cancer previously treated with trastuzumab and taxanes, significantly prolonging both PFS (9.6 months vs 6.4 months) and OS (30.9 months vs 25.1 months) while also achieving a higher ORR (43.6% vs 30.8%) with a more favorable toxicity profile. Although the DESTINY-Breast03 study^11^ reported that T-DM1 is less effective after failure of trastuzumab combined with pertuzumab, with an ORR of 34.2%, the efficacy of T-DM1 for Asians is still not very clear. In our phase II trial, 63.9% of the participants had previously received pyrotinib, and 41.7% had received both pyrotinib and pertuzumab. Promising clinical outcomes were found, with an ORR of 47.2% and a median PFS of 6.6 months. For HER2-positive metastatic breast cancer, T-DM1 is still an important choice.

HER2-positive breast cancer is still a heterogeneous disease, with intrinsically different subtypes of cancer cells. In our study, we identified 9 subtypes of cancer cells via scRNA-seq. CDK1+ cancer cells and TK1+ cancer cells, which have high levels of cell cycle activity, were abundant in resistant cases. MMP7+ cancer cells, enriched in genes associated with the humoral immune response, were abundant in sensitive cases. Preclinical studies^19^ have shown that increased activity of the CDK4/6 pathway mediates resistance to HER2-targeted therapies. Additionally, tumor cells that survive HER2 blockade retain high expression of cyclin D1.^18,25^ These studies suggest that cell cycle activity may be associated with resistance to HER2-targeted therapies. In our study, patients with high proportions of CDK1+ cancer cells in metastatic HER2 breast cancer patients who were validated in a neoadjuvant cohort had poor responses to T-DM1. These data suggest that the high cell cycle activity of cancer cells predicts a poor response to T-DM1 and that CDK4/6 inhibitors may reverse resistance,^26^ providing a new strategy for treating HER2-positive breast cancer. The tumor immune microenvironment plays an important role in the response to treatment in HER2-positive breast cancer.^27,28^ However, it remains unclear whether the tumor immune microenvironment could be a predictive biomarker in the context of ADC treatment. In our study, sensitive patients presented a strong antitumor CD8+ T-cell response, whereas resistant patients presented a relatively immunosuppressive microenvironment. Early activated CD8+ T cells were associated with a good response to T-DM1 treatment. Cell‒cell interaction analysis revealed that effective CD8+ T cells in sensitive patients had stronger interactions with macrophages than did those in resistant patients. Importantly, macrophages in sensitive cases are activated (e.g., M1 polarization) and favor the antitumor response. In our opinion, an activated immune microenvironment can be associated with the heterogeneity of cancer cells (e.g., MMP7+ cancer cells).

Several limitations still exist. First, genomic variations (e.g., *PIK3CA* mutations) in these tumor tissues were not detected, so the relationship between genomic variations and therapeutic efficacy remains unclear. Second, our findings derived from the scRNA-sequencing of limited samples of metastatic HER2-positive breast cancer patients were validated in a neoadjuvant cohort. Our findings should still be confirmed in metastatic breast cancer with large sample sizes. Third, the two samples after disease progression were obtained from two patients who experienced disease progression within very short intervals; these patients were likely to have primary resistance but not secondary resistance. Therefore, the 6 resistance cases in our study may reflect the characteristics of primary resistance. The tumor microenvironment of secondary resistance to T-DM1 should be determined in the future. Fourth, cell cycle activity, as a predictor of T-DM1 resistance, provides a treatment target to reverse T-DM1 resistance. CDK4/6 inhibitors combined with T-DM1 should be tested in the future.^26^

In summary, T-DM1 shows promising efficacy with a measurable safety profile in patients with metastatic HER2-positive breast cancer after failure of pyrotinib and/or trastuzumab plus pertuzumab. Importantly, low cell cycle activity and an activated immune microenvironment can predict the efficacy of T-DM1. Future clinical trials are warranted to confirm our findings.

## Materials and methods

### Study design and participants

This was a prospective single-arm, multicenter, phase Ⅱ trial (ClinicalTrials.gov identifier: NCT06125834). The study was approved by the institutional ethics committee of each enrolled center. Informed consent was obtained from all the participants before treatment. The key eligibility criteria were as follows: female patients aged ≥18 years, with HER2-positive metastatic breast cancer; measurable disease according to the Response Evaluation Criteria In Solid Tumors (RECIST) version 1.1; progressed disease after pyrotinib and/or trastuzumab combined with pertuzumab; an Eastern Cooperative Oncology Group (ECOG) status of 0 or 1; a left ventricular ejection fraction of 50% or more; and adequate organ and bone marrow function. The major exclusion criteria were prior treatment with T-DM1, clinical symptomatic central nerve system metastasis, a history of symptomatic congestive heart failure or severe cardiac arrhythmia requiring treatment, or a history of severe allergic reactions.

### Procedures

After enrollment, the participants received T-DM1 3.6 mg per kilogram of body weight intravenously every three weeks. Tumor assessments were performed every 6 weeks for the first 24 weeks and every 12 weeks thereafter. A complete or partial response should be confirmed 4 weeks later. Treatment-related adverse events were graded according to the National Cancer Institute Common Terminology Criteria for Adverse Events, version 4.03. Dose adjustment or discontinuation was defined according to the protocol until disease progression, unacceptable toxicity, patient withdrawal or death.

### Outcomes

The primary endpoint was the ORR according to RECIST 1.1, defined as the proportion of patients with the best complete or partial response. The secondary endpoints included PFS (time from the initiation of study treatment to disease progression or any-cause death), the disease control rate (DCR, proportion of patients with CR, PR or SD), the clinical benefit rate (CBR, proportion of patients with CR, PR, or SD ≥ 24 weeks), treatment-related adverse events, and potential biomarkers of T-DM1 resistance.

### Pathologic evaluation of tumor biopsies

Tumor specimens obtained through percutaneous biopsy were collected and processed for pathological assessment. The samples were fixed in 4% formaldehyde, paraffin-embedded, sectioned, and subsequently stained with hematoxylin and eosin (H&E) for histological examination.

### Tissue dissociation and preparation

Fresh tissue samples were stored in sCelLiveTM Tissue Preservation Solution (Singleron) on ice within 30 min after percutaneous biopsy. The tissues were subsequently washed three times with Hanks’ balanced salt solution (HBSS), finely minced, and then subjected to enzymatic digestion via 3 mL of sCelLive™ Tissue Dissociation Solution (Singleron) with the Singleron PythoN™ Tissue Dissociation System at 37 °C for 15 min. The cell suspension was collected and passed through a 40-micron sterile strainer, and red blood cells were lysed by adding GEXSCOPE^®^ Red Blood Cell Lysis Buffer (RCLB, Singleron) at a volume ratio of 1:2 (cell suspension: RCLB). This mixture was incubated at room temperature for 5–8 min. After centrifugation at 300 × *g* for 5 min at 4 °C, the supernatant was carefully aspirated, and the cell pellet was gently resuspended in phosphate-buffered saline (PBS). Finally, the samples were stained with Trypan blue, and cell viability was assessed microscopically.

### Library preparation for scRNA-seq

Single-cell suspensions at 2 × 10^5^ cells/mL in PBS (HyClone) were processed on a Singleron Matrix^®^ microwell chip. Barcoding beads were collected, and the bound mRNA was reverse transcribed to cDNA, followed by PCR amplification. The cDNA was fragmented and ligated with sequencing adapters, and libraries were constructed via the GEXSCOPE^®^ single-cell RNA library kit (Singleron).^29^ Libraries were diluted to 4 nM, pooled, and sequenced on an Illumina NovaSeq 6000 platform with 150 bp paired-end reads.

### Single-cell data analysis and processing

Raw sequencing reads were processed to derive gene expression profiles via CeleScope (version 1.5.2) (Singleron Biotechnologies) with standard parameters. Barcodes and UMIs were extracted and corrected from R1 reads, whereas R2 reads were trimmed of adapter sequences and poly A tails. Trimmed R2 reads were aligned to the GRCh38 (hg38) transcriptome via STAR (version 2.6.1b), and uniquely mapped reads were assigned to exons via FeatureCounts (version 2.0.1). Reads sharing the same cell barcode, UMI, and gene were aggregated to construct the gene expression matrix for downstream analysis.

The Seurat package (version 5.0.1) in R was used to analyze the scRNA-seq data. Low-quality cells, including those with over 10% mitochondrial content, more than 8000 genes, or fewer than 200 genes, as well as doublets, were filtered out, resulting in 80,239 cells for subsequent analysis. Data normalization was performed on the basis of raw UMI counts per cell, and 2000 highly variable genes were identified via the FindVariableFeatures function. The ScaleData function was applied to scale these genes, followed by principal component analysis (PCA) for dimensionality reduction and Harmony for batch effect correction. The FindNeighbors function calculates the shared nearest neighbor similarity in the batch-corrected cell-PC matrix, and the cell subpopulations are identified via the FindClusters function with a K-nearest neighbors (KNN) algorithm. Visualization of single-cell data was achieved via the uniform manifold approximation and projection (UMAP) algorithm. Differentially expressed genes for the cell clusters were identified via the COSine similarity-based marker gene identification (COSG) function.^30^

### Cell type abundance comparisons

We assessed differences in cell type abundance, including lineages and subclusters, via the Wilcoxon rank-sum test for pairwise comparisons and the Kruskal‒Wallis test for multiple group comparisons. *P* values are unadjusted for cell type abundance comparisons.

### Evaluation of cellular functions via single-cell data

GO and KEGG analyses were conducted via the clusterProfiler R package (version 4.8.3). The GSVA R package (version 1.48.3) was employed to conduct single-sample gene set enrichment analysis (ssGSEA), which quantifies pathway activity at single-cell resolution by calculating enrichment scores for predefined gene signatures. The algorithm computes the relative expression levels of target genes against background gene sets within individual cells, incorporating normalization against control gene sets to account for technical variability. The enrichment analysis was based on gene sets from the MsigDB database (http://www.gsea-msigdb.org/).

### Inference and analysis of cell‒cell communication

The CellChat R package (version 2.1.2) was employed for the quantitative assessment of intercellular communication networks.^31^ By utilizing network analytical techniques and pattern recognition, CellChat delineates the pivotal signaling pathways and cellular interactions that orchestrate diverse biological functions. It estimates the likelihood of cell‒cell interactions by integrating transcriptomic data with its repository of established signaling ligand‒receptor and cofactor interactions. Statistical comparisons between groups were performed via the Mann‒Whitney U test.

### RNA-seq analysis

Bulk RNA-seq data from HER2-positive breast cancer patients were obtained from the GEO database under accession number GSE243375.^20^ This analysis involved pretreatment tumor samples from 129 HER2-positive breast cancer patients who were treated with a combination therapy of T-DM1 and pertuzumab. Following normalization and imputation of the missing values in the RNA data, the GSVA R package (version 1.48.3) was used to perform ssGSEA for each sample on the basis of the expression profile. Gene sets composed of differentially expressed genes of cell subpopulations, as identified by the COSG function on the single-cell RNA sequencing data, were used for enrichment analysis.

### Statistical analysis

Simon’s two-stage design was used in this study. The null hypothesis of ORR was 20%, and the alternative hypothesis of ORR was 40%. A sample size of 36 achieved 80.211% power to detect a difference (P1--P0) of 0.2000 via a one-sided exact test with a significance level (alpha) of 0.0250. Efficacy assessment was performed both in the intention-to-treat (ITT, at least one cycle of study treatment) and efficacy-evaluable (at least one posttreatment evaluation) populations. The Clopper‒Pearson method was applied to calculate estimates of the ORR, DCR, and CBR and the corresponding 95% confidence intervals (CIs). The median durations of PFS and OS were estimated via the Kaplan‒Meier method.

For data from scRNA-seq and RNA-seq, continuous variables were characterized by the median and interquartile range. For these variables, we employed a two-tailed unpaired *t* test, Wilcoxon rank-sum test, or Kruskal‒Wallis test, as appropriate. A threshold of *P* < 0.05 was set to define statistical significance. The statistical analyses were performed via R software. Visualization of the outcomes was achieved through the ggplot2 R package (version 3.4.4) and the pheatmap R package (version 1.0.12).

## Supplementary information

Supplementary Materials

Study Protocol

Statistical Analysis Plan

CONSORT Checklist

## Data Availability

The raw sequence data reported in this paper have been deposited in the Genome Sequence Archive (Genomics, Proteomics & Bioinformatics 2021) of the National Genomics Data Center (Nucleic Acids Res 2022), China National Center for Bioinformation/Beijing Institute of Genomics, Chinese Academy of Sciences (GSA-Human: HRA009023), which is publicly accessible at https://ngdc.cncb.ac.cn/gsa-human.
