# Supplementary material for: Tumor microenvironment delineates differential responders to trastuzumab emtansine in HER2-positive metastatic breast cancer patients previously treated with pyrotinib: an exploratory biomarker analysis of a phase II study (NJMU-BC02)
Source: Signal Transduct Target Ther. 2025 Sep 29;10:318. doi: 10.1038/s41392-025-02409-2 (PMC12477294; doi:10.1038/s41392-025-02409-2)
Supplement: Supplementary file 1 — Supplementary Materials [file 41392_2025_2409_MOESM1_ESM.docx]

Supplementary Materials for

Tumor microenvironment delineates differential responders to trastuzumab emtansine in HER2-positive metastatic breast cancer patients previously treated with pyrotinib: an exploratory biomarker analysis of a phase II study (NJMU-BC02)

Hong Pan, Ji Wang, Yue Sun, Fanfan Li, Chang Sun, Mingduo Liu, Hong Xu, Jing Tao, Xinrui Mao, Cong Wang, Shui Wang, Wei Li, Qiang Ding, Wenbin Zhou

Correspondence to: zhouwenbin@njmu.edu.cn (W.Z.), dingqiang@njmu.edu.cn (Q.D.), liwei1218@njmu.edu.cn (W.L.).

**This PDF file includes:**

Figures. S1 to S3

Tables S1 to S3


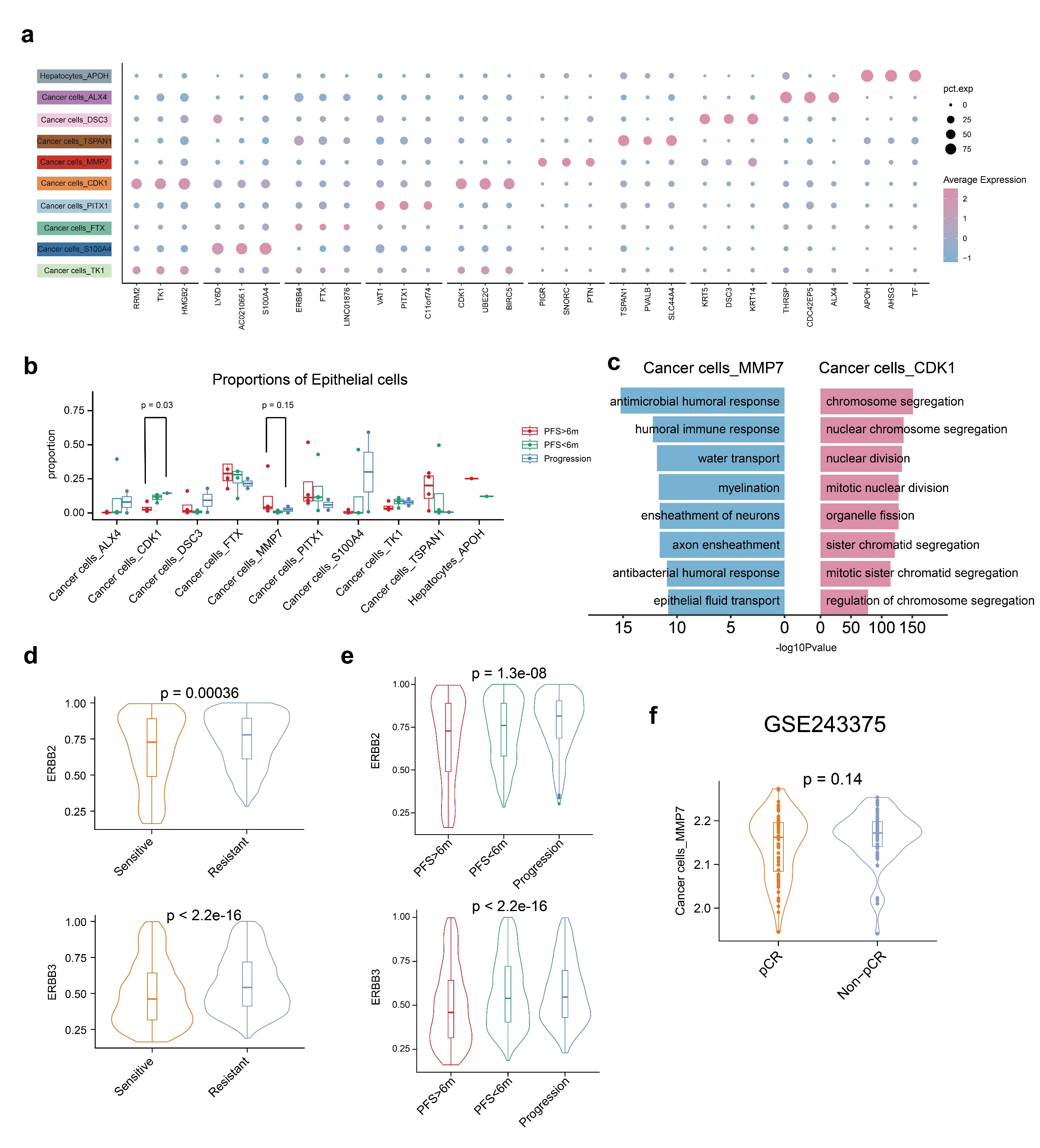


**Figure. S1.** Supplementary data related to the role of cell cycle activity in predicting the efficacy of trastuzumab emtansine. **a**, Dot plot of expression for genes defining epithelial cell clusters. **b**, Box plots showing the differences in proportions of epithelial cells among “PFS>6m”, “PFS<6m” and “Progression” groups. **c**, Bar plots showing the mean scores of GO pathways related to cancer cells_CDK1 and cancer cells_MMP7. **d**, Violin plots showing the differential expression of ERBB2 and ERBB3 between sensitive and resistant groups. **e**, Violin plots showing the differential expression of ERBB2 and ERBB3 among “PFS>6m”, “PFS<6m” and “Progression” groups. **f**, Violin plots showing the gene set enrichment score related to cancer cells_MMP7, based on RNA-seq data from GSE243375, calculated using ssGSEA. Data are represented as median and interquartile range (**d**, **e**, **f**). Significance determined by Wilcoxon rank-sum test (**d**), Kruskal-Wallis test (**b**, **e**) or two-tailed unpaired t-test (**f**). Significance was determined as P<0.05.


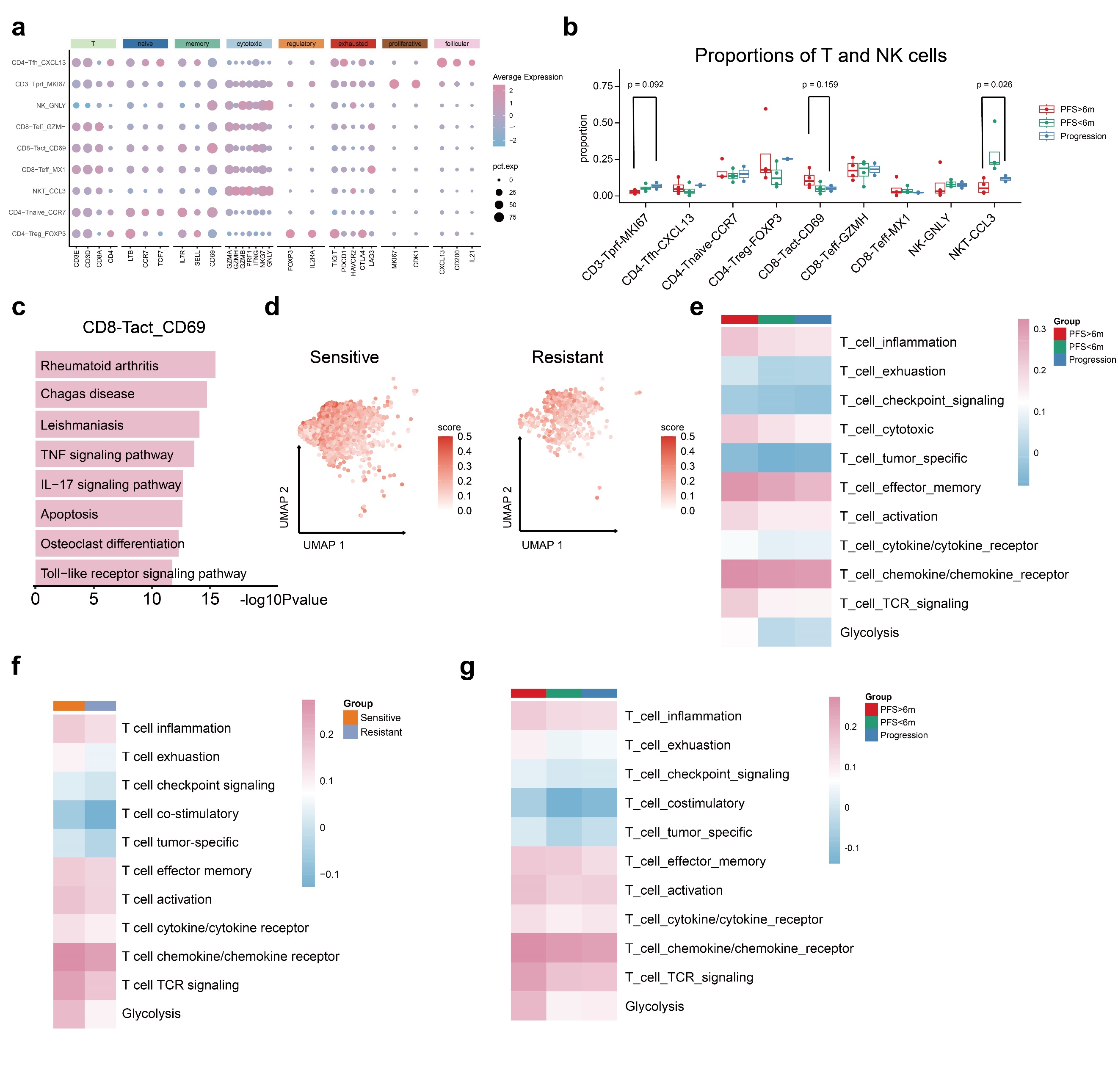


**Figure. S2.** Supplementary data related to the role of early activated CD8-Tact-CD69 cells in predicting the efficacy of trastuzumab emtansine. **a**, Dot plot of expression for genes defining T and NK cell clusters. **b**, Box plots showing the differences in proportions of T and NK cells among “PFS>6m”, “PFS<6m” and “Progression” groups. **c**, Bar plots showing the mean scores of KEGG pathways related to CD8-Tact-CD69. **d**, UMAP plots showing the T cell activation scores in CD8+T cells, calculated using ssGSEA. **e**, Heatmap of gene set enrichment scores for CD8+T cells among “PFS>6m”, “PFS<6m” and “Progression” groups, calculated using ssGSEA. **f**, Heatmap of gene set enrichment scores for CD4+T cells between sensitive and resistant groups, calculated using ssGSEA. **g**, Heatmap of gene set enrichment scores for CD4+T cells among “PFS>6m”, “PFS<6m” and “Progression” groups, calculated using ssGSEA. Data are represented as median and interquartile range (**b**). Significance determined by Kruskal-Wallis test (**b**). Significance was determined as P<0.05.


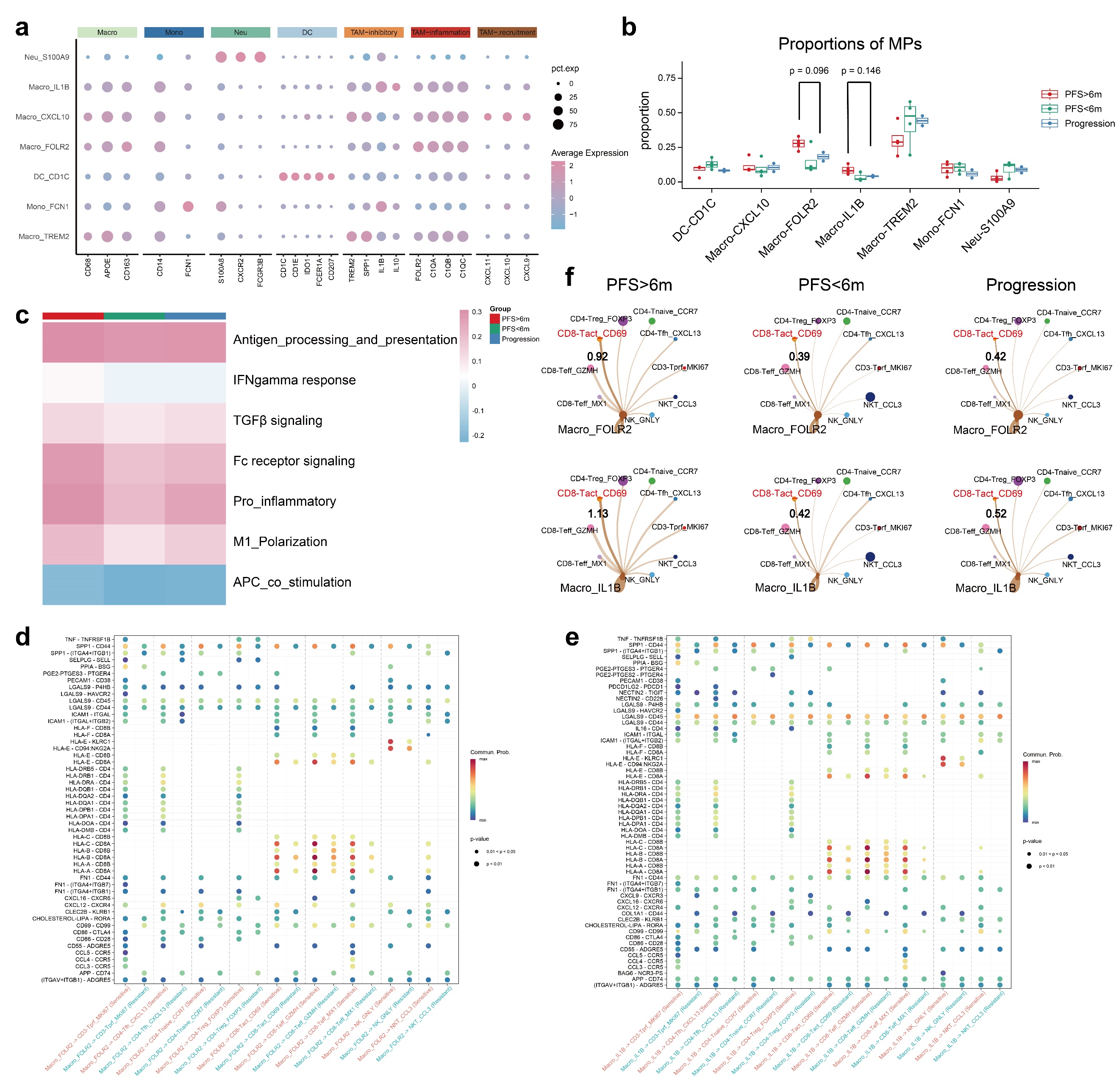


**Figure. S3.** Supplementary data related to the role of Macro-FOLR2 and Macro-IL1B cells in predicting the efficacy of trastuzumab emtansine. **a**, Dot plot of expression for genes defining MP clusters. **b**, Box plots showing the differences in proportions of MPs among “PFS>6m”, “PFS<6m” and “Progression” groups. **c**, Heatmap of gene set enrichment scores for macrophages among “PFS>6m”, “PFS<6m” and “Progression” groups, calculated using ssGSEA. **d**, Ligand-receptor interaction analysis between Macro-FOLR2 and T and NK cells. **e**, Ligand-receptor interaction analysis between Macro-IL1B and T and NK cells. **f**, Circle plots showing differential interaction strength between Macro-FOLR2 or Macro-IL1B and T and NK cells among “PFS>6m”, “PFS<6m” and “Progression” groups. Data are represented as median and interquartile range (**b**). Significance determined by Kruskal-Wallis test (**b**). Significance was determined as P<0.05.

**Table S1. The efficacy of trastuzumab emtansine in enrolled participants according to prior lines of therapy in the metastatic setting**

|  | **Intention-to-treat population (n = 36)** | | **Efficacy-evaluable population (n = 32)** | |
| --- | --- | --- | --- | --- |
|  | **≤1L** | **>1L** | **≤1L** | **>1L** |
| **Total** | 16 | 20 | 16 | 16 |
| **CR, n (%)** | 2 (12.5) | 0 (0) | 2 (12.5) | 0 (0) |
| **PR, n (%)** | 6 (37.5) | 9 (45.0) | 6 (37.5) | 9 (56.3) |
| **SD, n (%)** | 2 (12.5) | 5 (25.0) | 2 (12.5) | 5 (31.3) |
| **PD, n (%)** | 6 (37.5) | 2 (10.0) | 6 (37.5) | 2 (12.5) |
| **NE, n (%)** | 0 (0) | 4 (20.0) | 0 (0) | NA |
| **ORR, n (%)** | 8 (50.0) | 9 (45.0) | 8 (50.0) | 9 (56.3) |
| **DCR, n (%)** | 10 (62.5) | 14 (70.0) | 10 (62.5) | 14 (87.5) |
| **CBR, n (%)** | 8 (50.0) | 10 (50.0) | 8 (50.0) | 10 (62.5) |
| **mPFS (months)** | 5.2 | 5.2 | 5.2 | 5.95 |

**Abbreviations:** CR, complete response; PR, partial response; SD, stable disease; PD, progressive disease; NE, not evaluable; NA, not applicable; ORR, objective response rate; DCR, disease control rate; CBR, clinical benefit rate; mPFS, median progression-free survival.

**Table S2. Treatment-related adverse events**

|  | **T-DM1 (n=36)** | |
| --- | --- | --- |
|  | **Any grade AE, n (%)** | **Grade≥3 AE, n (%)** |
| **Diarrhoea** | 8 (22.2) | 1 (2.8) |
| **Vomiting** | 4 (11.1) | 0 (0) |
| **Hypokalaemia** | 1 (2.8) | 0 (0) |
| **Neutropenia** | 6 (16.7) | 0 (0) |
| **Nausea** | 7 (19.4) | 0 (0) |
| **Anaemia** | 2 (5.6) | 1 (2.8) |
| **ALT increased** | 3 (8.3) | 1 (2.8) |
| **AST increased** | 2 (5.6) | 1 (2.8) |
| **Thrombocytopenia** | 11 (30.6) | 4 (11.1) |

**Abbreviations:** T-DM1, trastuzumab emtansine; AE, adverse event; ALT, alanine aminotransferase; AST, aspartate aminotransferase.

**Table S3. Cell cycle associated genes**

| CDK1 | UBE2C | BIRC5 | PTTG1 | TOP2A | MKI67 | TPX2 | KIF2C | CDC20 | HMMR |
| --- | --- | --- | --- | --- | --- | --- | --- | --- | --- |
| CCNB1 | PBK | CDKN3 | MAD2L1 | NUF2 | NCAPG | CENPF | CCNA2 | ZWINT | HMGB2 |
| CDCA8 | KIF23 | TROAP | NDC80 | DLGAP5 | ANLN | PLK1 | AURKB | KIF20A | BUB1 |
| CDCA3 | SGO1 | CENPA | RRM2 | KIFC1 | NCAPH | DEPDC1 | CEP55 | CENPW | SPC25 |
| CENPE | GTSE1 | TK1 | NEK2 | CKAP2L | UBE2T | RACGAP1 | LMNB1 | PKMYT1 | ASPM |
| FOXM1 | TTK | ARHGAP11A | MYBL2 | TYMS | FAM83D | KIF11 | HJURP | KIF4A | CKAP2 |
| MELK | KNL1 | ATAD2 | ECT2 | TRIP13 | FBXO5 | SGO2 | STMN1 | RAD51AP1 | CKS1B |
| CENPU | MND1 | SMC4 | CENPN | KPNA2 | CENPK | CENPM | TACC3 | KIF20B | CKS2 |
| ESCO2 | SKA3 | PARPBP | CDCA5 | PIMREG | UBE2S | AURKA | ASF1B | TMPO | KIF14 |
| OIP5 | ORC6 | KNSTRN | SKA1 | FANCI | GAS2L3 | NCAPD2 | KIF15 | FAM111B | MCM10 |

The top 100 differentially expressed genes identified between Cancer cells_CDK1 and other epithelial subgroups using the COSG function were designated as the “cell cycle associated genes”.
