# Supplementary material for: Tumor microenvironment delineates differential responders to trastuzumab emtansine in HER2-positive metastatic breast cancer patients previously treated with pyrotinib: an exploratory biomarker analysis of a phase II study (NJMU-BC02)
Source: Signal Transduct Target Ther. 2025 Sep 29;10:318. doi: 10.1038/s41392-025-02409-2 (PMC12477294; doi:10.1038/s41392-025-02409-2)
Supplement: Supplementary file 2 — Study Protocol [file 41392_2025_2409_MOESM2_ESM.pdf]

Clinical Trial Protocol

Study Title: Tumor microenvironment delineating differential responders to trastuzumab emtansine in HER2-positive metastatic breast cancer previously treated by pyrotinib: an exploratory biomarker analysis of a phase II study (NJMU-BC02)

ClinicalTrials.gov ID: NCT06125834

Sponsor: The First Affiliated Hospital with Nanjing Medical University

Collaborators:

The Second Affiliated Hospital of Anhui Medical University

The First Affiliated Hospital of Soochow University

The Fourth Affiliated Hospital of Nanjing Medical University

Principal Investigator: Wenbin Zhou

Board Name: Ethics Committee of The First Affiliated Hospital with Nanjing Medical University, No.300, Guangzhou Road, Gulou District, Nanjing, China (Email: jsphkj@163.com)

Ethical approval number: 2023-SR-405

Study Duration: Oct 2023 - Mar 2025

Brief Summary:

The goal of this clinical trial is to learn about the efficacy and safety of trastuzumab emtansine (T-DM1) in the treatment of patients with advanced HER2-positive breast cancer after failure of TKIs or HP therapy. The main questions it aims to answer are:

- The objective response rate of patients receiving T-DM1 therapy with advanced HER2-positive breast cancer after failure of TKIs or HP therapy.
- The adverse events and prognosis of patients with advanced HER2-positive breast cancer who receive the T-DM1 therapy.
- Biomarkers with predictive significance for the efficacy of T-DM1, including tumor cell-related and immune microenvironment-related biomarkers.

Keywords

Breast cancer, HER2, trastuzumab emtansine, drug response biomarkers

Participants

Patients who had the history of HER2-positive breast cancer (either HER2 3+ in immunohistochemistry or HER2 amplification in fluorescent in situ hybridization) and underwent treatment failure of pyrotinib and/or trastuzumab plus pertuzumab.

Research objectives

(1) To investigate the efficacy and safety of T-DM1 for patients after failure of pyrotinib and/or trastuzumab plus pertuzumab.

(2) To explore biomarkers for predicting the efficacy of T-DM1 regimen among patients after failure of pyrotinib and/or trastuzumab plus pertuzumab.

Study Design

Study Type	Interventional, Single arm, Multicenter
Primary Purpose	Treatment
Study Phase	Phase 2
Interventional Study Model	Single Group Assignment
Number of Arms	1
Masking	None (Open Label)
Allocation	N/A
Enrollment	36

Arms and Interventions

**Arms**	**Assigned Interventions**
Experimental: T-DM1	Drug: T-DM1, 3.6 mg per kilogram of body weight, intravenously, every 3 weeks

Randomization method

Not applicable.

Inclusion Criteria

(1) Female patients at the age ≥ 18 years (for premenopausal or perimenopausal patients, a negative pregnancy test is required, and adequate contraceptive methods for women of childbearing potential during the treatment period);

(2) Patients with pathologically confirmed HER2-positive breast cancer (either HER2 3+ in immunohistochemistry or HER2 amplification in fluorescent in situ hybridization);

(3) Measurable disease according to the Response Evaluation Criteria In Solid Tumors (RECIST) version 1.1;
(4) Progressed disease after pyrotinib and/or trastuzumab combined with pertuzumab;
(5) An Eastern Cooperative Oncology Group (ECOG) status of 0 or 1;
(6) A left ventricular ejection fraction of 50% or more;
(7) Adequate organ and bone marrow function.

Exclusion Criteria
(1) Prior treatment with T-DM1;
(2) Clinically symptomatic central nerve system metastasis;
(3) A history of symptomatic congestive heart failure or serious cardiac arrhythmia requiring treatment;
(4) A history of severe allergic reaction.

Primary Endpoint
(1) Objective response rate (ORR)
Defined as the proportion of patients with best of complete response (CR) or partial response (PR).

Secondary Endpoints
(1) Disease control rate (DCR)
Defined as the proportion of patients with CR, PR or stable disease (SD).

(2) Clinical benefit rate (CBR)
Defined as the proportion of patients with CR, PR or SD ≥24 weeks.

(3) Progression-free survival (PFS)
Defined as time from the initiation of study treatment to disease progression or any-cause death.

(4) Safety and tolerability
Refer to the NCI-CTCAE v5.0 classification criteria for adverse events in clinical trials and oncology settings.

(5) Potential biomarkers of T-DM1 resistance

Statistical Analysis Plan

Simon's two-stage design was used in this study. The null hypothesis of ORR was 20%, and the alternative hypothesis of ORR was 40%. A sample size of 36 achieved 80.211% power to detect a difference (P1-P0) of 0.2000 using a one-sided exact test with a significance level (alpha) of 0.0250. Efficacy assessment was performed both in the intention-to-treat (ITT, at least one cycle of study treatment) and efficacy-evaluable (at least one post-treatment evaluation) population. The Clopper-Pearson method was applied to calculate estimates of ORR, DCR, CBR and the corresponding 95% confidence intervals (CIs). The median durations of PFS and OS were estimated by the Kaplan-Meier method.

For data from scRNA-seq and RNA-seq, continuous variables were characterized by the means ± standard deviations (SDs). For these variables, we employed a two-tailed unpaired t-test, Wilcoxon rank-sum test, or Kruskal-Wallis test as appropriate. A threshold of $P < 0.05$ was set to define statistical significance. The statistical analyses were performed utilizing R software. Visualization of the outcomes was achieved through the ggplot2 R package (version 3.4.4) and the pheatmap R package (version 1.0.12).

The software, packages, and methods used for the analysis of the scRNA-seq and RNA-seq data are described in the MATERIALS AND METHODS section of this article.

Drug Information

Name: Trastuzumab Emtansine (T-DM1)

Specification: 100 mg or 160 mg per vial

Approach of Administration: IVD

Manufacturer: Roche Schweiz

Appendix 1

Criteria for Evaluating the Severity of Adverse Events

Refer to the NCI-CTCAE v5.0 classification criteria for adverse events in clinical trials and oncology settings. URL: https://ctep.cancer.gov/protocolDevelopment/electronic_applications/ctc.htm)

If there are adverse events that are not listed in the NCI-CTCAE v5.0 table, the following criteria may be used:

**Grade**	**Clinical description of severity**
1	Mild; no clinical symptoms or mild clinical symptoms; clinical or laboratory abnormalities only; no treatment is required
2	Moderate; requiring minor, local, or non-invasive treatment; Age-appropriate activities of daily living (ADL) with tools for cooking, shopping, making phone calls, counting money, etc
3	Severe illness or medically serious symptoms that are not life-threatening for the time being; resulting in hospitalization or prolonged hospital stay; resulting in disability; self-care ADL is limited. Self-care in daily life refers to: bathing, dressing, undressing, eating, going to the bathroom, taking medicine, etc., but not bedridden
4	Life-threatening; urgent treatment is required
5	Death due to adverse events

Appendix 2

Research management

(1) Data Review and Monitoring

The Clinical report form is filled out by the investigator, and each selected subject must complete the form. After the clinical monitor reviews the completed Clinical report form, the first page will be handed over to the data administrator for data entry and management. Data entry and management are the responsibility of the designated data administrator of the Statistics Department. Data administrators compile data entry procedures for data entry and management. To ensure the accuracy of the data, two data administrators should independently double-entry and proofread. For the questions in the report form, the data administrator will generate a Question Answer Form (DRQ) and send questions to the investigator through the clinical monitor. The researcher should answer and return it as soon as possible. Modify, confirm and enter, and reissue DRQ if necessary. After reviewing and ensuring that the established database is correct, the principal investigator and statistical analyst will lock the data, and the locked data files will not be changed.

(2) Image Data Measurement

Imaging data (MRI data, ultrasound data) are unified and jointly measured by two experienced professional physicians. The collection and follow-up of scale information are completed by special personnel according to a unified standardized method.

(3) Data Security Audits

Clinical studies will establish corresponding data security monitoring plans based on the size of the risks. All adverse events will be accurately recorded, appropriately handled, and tracked until resolved or the condition stabilizes. Serious adverse events and unexpected events will be reported to the ethics committee, regulatory authorities, sponsors, and drug regulatory agencies in a timely manner according to regulations. The principal investigators will periodically review all adverse events in an accumulative manner, and if necessary, a researcher meeting will be convened to assess the risks and benefits of the study. Studies with risks higher than the minimum level will arrange for independent data monitors to conduct monitoring on the research data, while high-risk studies will establish an independent data safety monitoring committee to monitor the accumulated safety and efficacy data and make recommendations on whether the study should continue.
