# Supplementary material for: Tumor microenvironment delineates differential responders to trastuzumab emtansine in HER2-positive metastatic breast cancer patients previously treated with pyrotinib: an exploratory biomarker analysis of a phase II study (NJMU-BC02)
Source: Signal Transduct Target Ther. 2025 Sep 29;10:318. doi: 10.1038/s41392-025-02409-2 (PMC12477294; doi:10.1038/s41392-025-02409-2)
Supplement: Supplementary file 3 — Statistical Analysis Plan [file 41392_2025_2409_MOESM3_ESM.pdf]

Pan H, et al. Tumor microenvironment delineating differential responders to trastuzumab emtansine in HER2-positive metastatic breast cancer previously treated by pyrotinib: an exploratory biomarker analysis of a phase II study (NJMU-BC02). Signal Transduction and Targeted Therapy.

Statistical Analysis Plan

Simon's two-stage design was used in this study. The null hypothesis of ORR was 20%, and the alternative hypothesis of ORR was 40%. A sample size of 36 achieved 80.211% power to detect a difference (P1-P0) of 0.2000 using a one-sided exact test with a significance level (alpha) of 0.0250. Efficacy assessment was performed both in the intention-to-treat (ITT, at least one cycle of study treatment) and efficacy-evaluable (at least one post-treatment evaluation) population. The Clopper-Pearson method was applied to calculate estimates of ORR, DCR, CBR and the corresponding 95% confidence intervals (CIs). The median durations of PFS and OS were estimated by the Kaplan-Meier method.

For data from scRNA-seq and RNA-seq, continuous variables were characterized by the means ± standard deviations (SDs). For these variables, we employed a two-tailed unpaired t-test, Wilcoxon rank-sum test, or Kruskal-Wallis test as appropriate. A threshold of $P < 0.05$ was set to define statistical significance. The statistical analyses were performed utilizing R software. Visualization of the outcomes was achieved through the ggplot2 R package (version 3.4.4) and the pheatmap R package (version 1.0.12).

The software, packages, and methods used for the analysis of the scRNA-seq and RNA-seq data are described in the MATERIALS AND METHODS section of this article.
